# Comorbidity, malnutrition, and their additive effect on prognosis in cancer patients: a multicenter cohort study

**DOI:** 10.1016/j.jnha.2026.100960

**Published:** 2026-08-25

**Authors:** Kaijia Zhao, Huanyu Hu, Hongzhen Du, Caiyan Zhang, Yeze Jia, Xiaoya Wang, Xuanhui Wu, Yan Guo, Yijing Zhai, Mingyang Fu, Anqi Wang, Chunhua Song, Hanping Shi, Zengning Li, the Investigation on Nutrition Status and Clinical Outcome of Common Cancers (INSCOC) Group

**Affiliations:** a School of Public Health, Hebei Medical University, Shijiazhuang, China; b Department of Clinical Nutrition, The First Hospital of Hebei Medical University, Shijiazhuang, Hebei, China; c Hebei Province Key Laboratory of Nutrition and Health, Shijiazhuang, Hebei, China; d Department of Clinical Nutrition/Hebei Key Laboratory of Stomatology/Hebei Technology Innovation Center of Oral Health, School and Hospital of Stomatology, Hebei Medical University, Shijiazhuang, China; e Department of Epidemiology and Statistics, College of Public Health, Zhengzhou University, Zhengzhou, China; f Department of Clinical Nutrition, Beijing Shijitan Hospital, Capital Medical University, Beijing, China; g State Market Regulation, Key Laboratory of Cancer FSMP for State Market Regulation, Beijing, China

**Keywords:** Cancer, Malnutrition, Comorbidity, Prognosis, Older patients

## Abstract

**Background:**

With population aging and the rising prevalence of chronic disease worldwide, comorbidity is prevalent among patients with cancer. However, the relationship between comorbidity and malnutrition, as well as their combined impact on prognosis remains poorly understood.

**Methods:**

We analyzed data of 12,947 cancer patients from a large-scale multicenter cohort study. Comorbidity burden was quantified using the modified age-adjusted Charlson Comorbidity Index (mACCI). Malnutrition was diagnosed using the Global Leadership Initiative on Malnutrition (GLIM). Clinical outcomes included overall survival, 30-day mortality, prolonged length of hospital stay (LOS), and increased hospitalization costs. Multivariable regression models were employed.

**Results:**

10,673 (82.4%) patients were diagnosed with malnutrition, and 7,982 (62.0%) patients had a high comorbidity burden (mACCI ≥ 2). The comorbidity burden and malnutrition were significantly correlated only in patients with stage I disease (OR = 1.60, 95% CI: 1.13–2.25). Some comorbidities, including anemia, non-stroke cardiovascular/cerebrovascular disease, chronic kidney disease, chronic pulmonary disease, and diabetes, had specific associations with the phenotypic or etiological characteristics of malnutrition (all *p* < 0.05). Malnutrition was an independent prognostic factor for all four adverse outcomes (all *p* < 0.05), and high comorbidity burden was an independent prognostic factor for prolonged LOS and increased costs (all *p* < 0.05). Patients with both high comorbidity burden and malnutrition exhibited a higher risk of 30-day mortality (OR = 4.58, 95% CI: 1.30–29.1), prolonged LOS (OR = 1.33, 95% CI: 1.12–1.58), and increased costs (OR = 1.35, 95% CI: 1.14–1.60) compared with those with low comorbidity burden and well-nourished status.

**Conclusion:**

The relationship between comorbidity burden and malnutrition was stage-dependent, and their coexistence had an additive effect on the prognosis of cancer patients. These findings highlight the importance of integrating comorbidity assessment into the nutritional management of cancer patients, particularly in early-stage disease.

## Highlights

• The association between a high comorbidity burden and malnutrition is stage-dependent, with statistical significance observed only in stage I cancer patients.
• Different comorbidities show distinct associations with the phenotypic and etiologic characteristics of malnutrition.
• The coexistence of a high comorbidity burden and malnutrition is associated with the poorest clinical outcomes, supporting the value of joint assessment for prognostic stratification.

## 1 Introduction

Approximately 40%–80% of patients with cancer suffer from malnutrition [1,2]. It is an independent predictor of adverse outcomes, including shorter overall survival (OS) [[3], [4], [5]]. 20% of cancer deaths are due to malnutrition rather than the cancer itself [6]. Therefore, accurate identification of patients at high risk of malnutrition remains a challenge in oncologic supportive care. Previous studies have focused on cancer-related factors such as cancer site, stage, and treatment [7], while research on the role of comorbidity, which is highly prevalent in this population, remains underexplored.

With population aging and the rising prevalence of chronic diseases worldwide, comorbidity has become increasingly common among patients with cancer. In this study, we follow the classic definition proposed by Feinstein et al. [8] and define comorbidity as a chronic disease that coexists with cancer (excluding cancer). Previous research has shown that more than half of cancer patients have at least one comorbidity [9]. Comorbidities may affect the nutritional status through chronic inflammation, metabolic disorders, and impaired nutrient absorption [10,11]. Emerging evidence also suggests that comorbidity is associated with nutritional vulnerability or skeletal muscle loss in patients with cancer [12,13]. However, to translate this broad association into clinically actionable strategies, a deeper understanding of how specific comorbidities are linked to different dimensions of malnutrition is required. The Global Leadership Initiative on Malnutrition (GLIM) [14], with its phenotypic and etiologic criteria, provides a framework for this. Investigating these specific associations is not only important for achieving personalized nutritional management but also has prognostic value. In clinical practice, comorbidity and malnutrition can impose a double burden on patients. Although their respective adverse effects on prognosis have been extensively validated [5,13], their combined effects on critical outcomes have not been adequately evaluated.

Therefore, using data from a large-sample, multicenter prospective cohort, this study aimed to systematically investigate (1) the association between comorbidity burden and malnutrition in cancer patients, (2) the specific links between comorbidities and the phenotypic/etiologic characteristics of malnutrition, and (3) the independent and combined prognostic implications of comorbidity burden and malnutrition.

## 2 Materials and methods

### 2.1 Study design and population

This study was based on a large multicenter prospective cohort, the Investigation on Nutrition Status and its Clinical Outcome of Common Cancers (INSCOC) project (Chinese Clinical Trial Registry, No. ChiCTR1800020329). The study protocol has been described previously [15,16]. The present analysis used data collected between January 2013 and December 2023. Eligible patients met all of the following criteria: (1) a confirmed diagnosis of malignancy, (2) age ≥18 years, and (3) a complete baseline comorbidity assessment. Patients who were critically ill, unable to cooperate, or were hospitalized with psychiatric disorders or memory impairment and therefore could not provide reliable information were excluded. Finally, 12,947 patients were included. The detailed screening process is shown in Figure S1.

### 2.2 Ethics

The project was approved by the Ethics Committees of the registration hospital (Beijing Shijitan Hospital) and all other participating institutions. The study was conducted in accordance with the ethical principles of the Declaration of Helsinki and its later amendments. All participants provided written informed consent within 48 h of hospital admission, prior to their inclusion in the study and any data collection.

### 2.3 Data collection

Baseline data were collected within 48 h of admission by trained nutritionists using a validated case report form. All baseline data were extracted from the electronic medical record system, except for nutritional assessment data obtained through interviews and anthropometric measurements, as well as survival status. The collected data included:

1) **Demographic information**: including sex and age.
2) **Tumor characteristics:** including cancer site (lung and bronchus; esophagus; stomach; liver and intrahepatic bile ducts; pancreas; colon and rectum; breast, ovary, uterus, and cervix; kidney and bladder; nasopharynx; and others, including brain, other nervous system, and hematological malignancies), TNM stage (assessed according to the Tumor-Node-Metastasis staging system based on the American Joint Committee on Cancer 8th edition guidelines [17]), and type of anticancer treatment (including no anticancer treatment, surgery, chemoradiotherapy, surgery with chemo/radiotherapy, and others).
3) **Nutrition-related assessment indicators.** Nutritional status was assessed using anthropometric measurements, biochemical indicators, and interview-based information. Anthropometry included height, weight, calf circumference (CC), mid-arm circumference (MAC), and triceps skinfold thickness (TSF, mm). Body mass index (BMI) was calculated as weight (kg)/height (m²). Mid-arm muscle circumference (MAMC, cm) was calculated as: MAC (cm) − (0.314 × TSF [mm]). Data on non-volitional weight loss, reduced food intake, and gastrointestinal symptoms suggesting impaired absorption were obtained through structured interviews. Biochemical indicators included white blood cell count, neutrophil count, lymphocyte count, C-reactive protein (CRP), and albumin. The neutrophil-to-lymphocyte ratio (NLR) was calculated as neutrophil count divided by lymphocyte count.
4) **Outcome measures:** four outcomes were assessed: overall survival (OS), 30-day mortality, prolonged length of hospital stay (LOS), and increased hospitalization costs. OS was defined as the time from admission to death from any cause. Follow-up for patients began at admission. Study investigators collected outcome information through review of outpatient/inpatient records or through face-to-face and telephone interviews with patients or their families. The first follow-up was conducted at 30 days after admission to determine 30-day outcomes. Subsequent follow-ups were conducted annually until death, loss to follow-up, or the end of the study period on December 31, 2023. For patients who remained alive, survival time was censored at the date of their last known contact or on December 31, 2023, whichever occurred first. Patients who could not be contacted after continuous attempts over six months were defined as lost to follow-up, and their data were censored at the date of last confirmed survival. In line with previous studies from this cohort [18], prolonged LOS was defined as a hospital stay of ≥ 14 days, and increased hospitalization cost was defined as costs at or above 20,000 RMB.

### 2.4 Assessment of comorbidity burden

As noted above, we followed the classic definition proposed by Feinstein [8], defining comorbidity as any chronic disease coexisting with but distinct from cancer. Comorbidity was assessed using 17 chronic diseases collected in the study, including cardiovascular/cerebrovascular diseases (stroke, myocardial infarction, coronary heart disease, hypertension), gastrointestinal/hepatic diseases (chronic pancreatitis, ulcerative colitis, Crohn's disease, chronic biliary tract disease, chronic hepatitis, cirrhosis), endocrine/metabolic diseases (diabetes, hyperthyroidism, hypothyroidism), chronic pulmonary disease (chronic obstructive pulmonary disease), connective tissue disease (systemic lupus erythematosus), renal diseases (chronic kidney disease, dialysis), and anemia.

To quantify comorbidity burden, we used the modified Age-Adjusted Charlson Comorbidity Index (mACCI) as described by Koseki and his colleagues [19], which is based on the ACCI developed by Charlson et al. [20] with the cancer-related weight removed. The theoretical basis and weighting system detailed in Table S1 are derived from the original framework and subsequent updates [[20], [21], [22], [23]]. This modification ensured that the comorbidity assessment was independent of the cancer itself, thereby preventing potential collinearity with variables like tumor stage in subsequent regression models. Based on the median mACCI score in this cohort, patients were classified into low (mACCI score < 2) and high (mACCI score ≥ 2) comorbidity burden groups for ease of clinical interpretation and to facilitate subgroup analysis.

### 2.5 Diagnosis of malnutrition

Malnutrition was diagnosed according to the GLIM criteria, requiring the presence of at least one phenotypic criterion (non-volitional weight loss, low BMI, or low muscle mass) and one etiologic criterion (reduced food intake/assimilation or disease burden/inflammation). Detailed diagnostic parameters and thresholds are presented in Table S2. For low muscle mass, CC and MAMC were applied using fixed sex-specific cut-offs.

Following the methodological approach of relevant studies [24], nutritional status was diagnosed using a direct application of the GLIM criteria rather than the clinically recommended two-step approach of screening and subsequent diagnosis. This methodological choice reflects the difference between the study objective and routine clinical workflow. Nutritional risk screening tools are mainly used in busy clinical settings for rapid triage and healthcare resource allocation. This cohort study included complete data and aimed to provide the most accurate scientific assessment rather than to manage clinical resources. Moreover, direct diagnosis could avoid bias caused by missed diagnoses of false-negative cases due to the limited sensitivity of screening tools [25], thereby ensuring a robust foundation for our statistical analyses.

### 2.6 Statistical analysis

The sample size was determined by the existing cohort. To evaluate the stability of the multivariable models and the reliability of the findings, we calculated the events per variable (EPV) for each model. The EPV exceeded the conventional threshold of 10 [26,27] for all models except for the 30-day mortality model, which had an EPV of approximately 8. The results of Vittinghoff et al. suggest that robust estimates are achievable with an EPV > 5 in large samples [28]. Therefore, the 30-day mortality model was considered informative, although its results should be interpreted with caution.

Continuous variables were presented as mean ± standard deviation (Mean ± SD) or median with interquartile range [M (IQR)] depending on their distribution. Comparisons between well-nourished and malnourished patients were performed using the unpaired t-test or the Mann–Whitney U test. Categorical variables were presented as numbers and percentages, and compared using the chi-square test or Fisher’s exact test. To quantify the association between comorbidity burden and malnutrition, we constructed sequential multivariable logistic regression models. The analysis proceeded as follows: Model 1 was unadjusted. Model 2 adjusted for age and sex. Model 3 further adjusted for key oncologic variables based on model 2, including cancer site, stage, and treatment. To explore heterogeneity in this association across cancer stages, we investigated the interaction between mACCI and different cancer stages and performed subgroup analyses stratified by cancer stage (I, II, III, and IV).

To explore the associations between specific comorbidities and characteristics of malnutrition, we performed multinomial logistic regression analyses. Malnourished patients were categorized into mutually exclusive groups based on the GLIM criteria, which served as the categorical dependent variable. Four phenotype profiles were defined: (1) weight loss only; (2) low muscle mass only; (3) low BMI only; and (4) mixed phenotype (presence of at least two phenotypic criteria). Similarly, three etiology profiles were categorized: (1) reduced food intake/assimilation only; (2) inflammation only; and (3) mixed etiology (presence of both reduced food intake/assimilation and inflammation). Well-nourished patients served as the reference group. The analysis was conducted in two stages. We first visualized unadjusted associations by plotting odds ratios (ORs) from univariate models in heatmaps. The OR values reflect the direction and strength of the unadjusted association. Subsequently, multivariable models adjusting for age, sex, and oncologic covariates were constructed to estimate independent associations, presented as ORs with 95% confidence intervals (CIs). Patients with connective tissue disease (*n* = 5) and dialysis (*n* = 11) were excluded from regression analyses in this section due to their low prevalence.

Finally, the independent and combined effects of comorbidity burden and malnutrition on clinical outcomes were evaluated. We used multivariable Cox proportional hazards model and multivariable logistic regression model to analyze their independent impact on OS and other binary outcomes respectively. To investigate their combined effects, patients were divided into four subgroups: (1) low comorbidity burden and well-nourished, serving as the reference group; (2) high comorbidity burden and well-nourished; (3) low comorbidity burden and malnourished; (4) high comorbidity burden and malnourished. Kaplan-Meier curves and bar charts were used to present the outcomes of patients in the four subgroups, and univariate statistical tests were performed. Multivariable Cox proportional hazards and logistic regression models were used to evaluate their combined impact on the outcomes.

A key methodological consideration was the adjustment for the important confounding factor of age. The mACCI scoring system assigns points in broad categories for patients aged 50 years and older, and such crudeness may lead to information loss and residual confounding. Therefore, we included continuous age as a separate covariate in all multivariable models to more precisely isolate the independent effects of the comorbidity burden. The statistical stability of this method was ensured by assessing multicollinearity between age and the mACCI score. All variance inflation factor (VIF) values were below the conventional threshold of 5, indicating that our modeling method was robust [29]. Additionally, we conducted a sensitivity analysis by omitting the continuous variable age, which revealed a significantly stronger association between mACCI and malnutrition (Table S3). This indicates the presence of residual confounding where the mACCI score absorbed part of the effect of age. Therefore, our primary modeling strategy could be used for a more accurate and less biased assessment of the effect of comorbidity.

Additionally, to assess the robustness of our findings regarding the mACCI, a second sensitivity analysis was conducted by replacing the binary mACCI variable with its original continuous form in the prognostic models.

In this study, all statistical analyses were performed using R software (Version 4.5.2). A two-sided P value less than 0.05 was considered statistically significant.

## 3 Results

### 3.1 Demographic and clinical characteristics

Of the 12,947 included patients, 10,673 (82.4%) were diagnosed with malnutrition (Table 1). Patients with malnutrition were older (median: 60 vs. 57 years), more likely to be female (43.4% vs. 33.4%), and had a greater proportion of digestive system tumors (48.5% vs. 40.4%), advanced disease (Stage IV: 46.1% vs. 42.0%), and high comorbidity burden (62.8% vs. 56.4%) (all *p* < 0.001). Conversely, cardiovascular disease was more common in the well-nourished group (28.7% vs. 25.9%; *p* = 0.006). As expected, patients with malnutrition exhibited significantly lower BMI, CC, and MAMC (all *p* < 0.001). Furthermore, hypoalbuminemia (<35 g/L) was substantially more common in this group (22.4% vs. 10.2%; *p* <  0.001). Regarding clinical outcomes, malnutrition was associated with shorter overall survival (OS), longer length of stay (LOS), and higher 30-day mortality (all *p* < 0.001). No significant difference was observed in total hospitalization costs (*p* = 0.469).

**Table 1:** Baseline characteristics of patients.

Characteristic	All patients *N* = 12,947^1^	GLIM-defined malnutrition		P-value^2^
		Well-nourished patients *N* = 2,274^1^	Malnourished patients *N* = 10,673^1^	
**General information**				
Age, years	59 (51, 66)	57 (50, 65)	60 (51, 67)	**<0.001**
Female	5,397 (41.7%)	760 (33.4%)	4,637 (43.4%)	**<0.001**
**Cancer-related Information**				
Cancer site				**<0.001**
Digestive system solid tumor	6,095 (47.1%)	919 (40.4%)	5,176 (48.5%)	
Hematological malignancy	530 (4.1%)	84 (3.7%)	446 (4.2%)	
Non-digestive solid tumor	5,776 (44.6%)	1,177 (51.8%)	4,599 (43.1%)	
Others	546 (4.2%)	94 (4.1%)	452 (4.2%)	
Cancer Stage				**<0.001**
Stage I	1,891 (14.6%)	379 (16.7%)	1,512 (14.2%)	
Stage II	1,537 (11.9%)	258 (11.3%)	1,279 (12.0%)	
Stage III	3,641 (28.1%)	681 (29.9%)	2,960 (27.7%)	
Stage IV	5,878 (45.4%)	956 (42.0%)	4,922 (46.1%)	
Current Cancer-related Treatment				**<0.001**
No treatment	2,757 (21.3%)	548 (24.1%)	2,209 (20.7%)	
Surgery only	1,521 (11.7%)	212 (9.3%)	1,309 (12.3%)	
Chemoradiotherapy only	5,267 (40.7%)	921 (40.5%)	4,346 (40.7%)	
Surgery with chemo/radio	3,354 (25.9%)	589 (25.9%)	2,765 (25.9%)	
Other treatments	48 (0.4%)	4 (0.2%)	44 (0.4%)	
**Comorbidity**				
mACCI ≥2	7,982 (62.0%)	1,283 (56.4%)	6,699 (62.8%)	**<0.001**
Comorbidity Categories				
Cardiovascular/Cerebrovascular	3,420 (26.4%)	653 (28.7%)	2,767 (25.9%)	**0.006**
Gastrointestinal/Hepatic	1,293 (10.0%)	232 (10.2%)	1,061 (9.9%)	0.706
Endocrine/Metabolic	1,759 (13.6%)	307 (13.5%)	1,452 (13.6%)	0.895
Chronic pulmonary disease	278 (2.1%)	43 (1.9%)	235 (2.2%)	0.423
Connective tissue disease	5 (0.04%)	2 (0.09%)	3 (0.03%)	0.214
Renal Disease	74 (0.6%)	18 (0.8%)	56 (0.5%)	0.125
Anemia	546 (4.2%)	56 (2.5%)	490 (4.6%)	**<0.001**
**Anthropometric and Laboratory Measurements**				
BMI, kg/m²	22.3 (20.0, 24.6)	25.2 (23.4, 27.3)	21.7 (19.6, 23.9)	**<0.001**
Calf circumference, cm	33.0 (30.5, 35.0)	36.0 (35.0, 38.0)	32.0 (30.0, 34.0)	**<0.001**
Mid-arm muscle circumference, cm	21.6 (19.4, 23.6)	24.8 (23.7, 26.3)	20.8 (18.9, 22.5)	**<0.001**
Serum Albumin				**<0.001**
<35 g/L	2,503 (20.3%)	218 (10.2%)	2,285 (22.4%)	
≥35 g/L	9,853 (79.7%)	1,927 (89.8%)	7,926 (77.6%)	
**Outcomes**				
Overall Survival, days	774 (431, 1,253)	836 (533, 1,341)	755 (413, 1,232)	**<0.001**
Length of Hospital Stay, days	10 (6, 18)	10 (6, 16)	11 (6, 18)	**<0.001**
Total Hospitalization Cost, ¥	16,522 (9,236, 37,110)	15,831 (8,778, 39,224)	16,670 (9,341, 36,890)	0.469
30-day mortality	103 (0.8%)	6 (0.3%)	97 (0.9%)	**<0.001**

Abbreviations: GLIM, Global leadership initiative on Malnutrition; mACCI: modified Age-adjusted Charlson Comorbidity Index; BMI: Body Mass Index.

1 Median (Q1, Q3); n (%).

2 Wilcoxon rank sum test; Pearson's chi-squared test; Fisher's exact test.

### 3.2 The association between comorbidity burden and malnutrition was stage-dependent

In the unadjusted model, a high comorbidity burden was significantly associated with malnutrition (OR = 1.30, 95% CI: 1.19–1.43). This association was no longer statistically significant after adjustment for confounders (Table 2). Given a significant statistical interaction between comorbidity burden and cancer stage (*p* for interaction = 0.006), we performed stage-stratified analyses. After adjustment, a high comorbidity burden was independently associated with malnutrition only in stage I patients (OR = 1.60, 95% CI: 1.13–2.25). No significant association was detected among patients with stage II, III or IV cancer.

**Table 2:** Multivariable and subgroup analyses of the association between comorbidity burden and malnutrition.

Characteristic	Overall population (*N* = 12947)			Subgroup analysis by cancer stage (using Model 4)			
	Model 1	Model 2	Model 3	Stage I (*n* = 1891)	Stage II (*n* = 1537)	Stage III (*n* = 3641)	Stage IV (*n* = 5878)
Comorbidity Burden, OR (95% CI)							
mACCI＜2	Ref.	Ref.	Ref.	Ref.	Ref.	Ref.	Ref.
mACCI≥2	1.30 (1.19, 1.43)	1.07 (0.94, 1.23)	1.06 (0.92, 1.21)	1.60 (1.13, 2.25)	1.37 (0.90, 2.10)	0.89 (0.69, 1.14)	0.99 (0.80, 1.22)
P-value	**<0.001**	0.316	0.412	**0.007**	0.142	0.345	0.918

Model 1: Crude model.

Model 2: Adjusted for age, sex.

Model 3: Adjusted for age, sex, cancer site, cancer stage, and cancer-related treatment.

Model 4: Adjusted for age, sex, cancer site, and cancer-related treatment.

*p* for interaction between Comorbidity Burden (mACCI ≥2) and Cancer Stage = 0.006.

### 3.3 Associations between comorbidities and phenotypic/etiologic characteristics of malnutrition

**Fig. 1:**
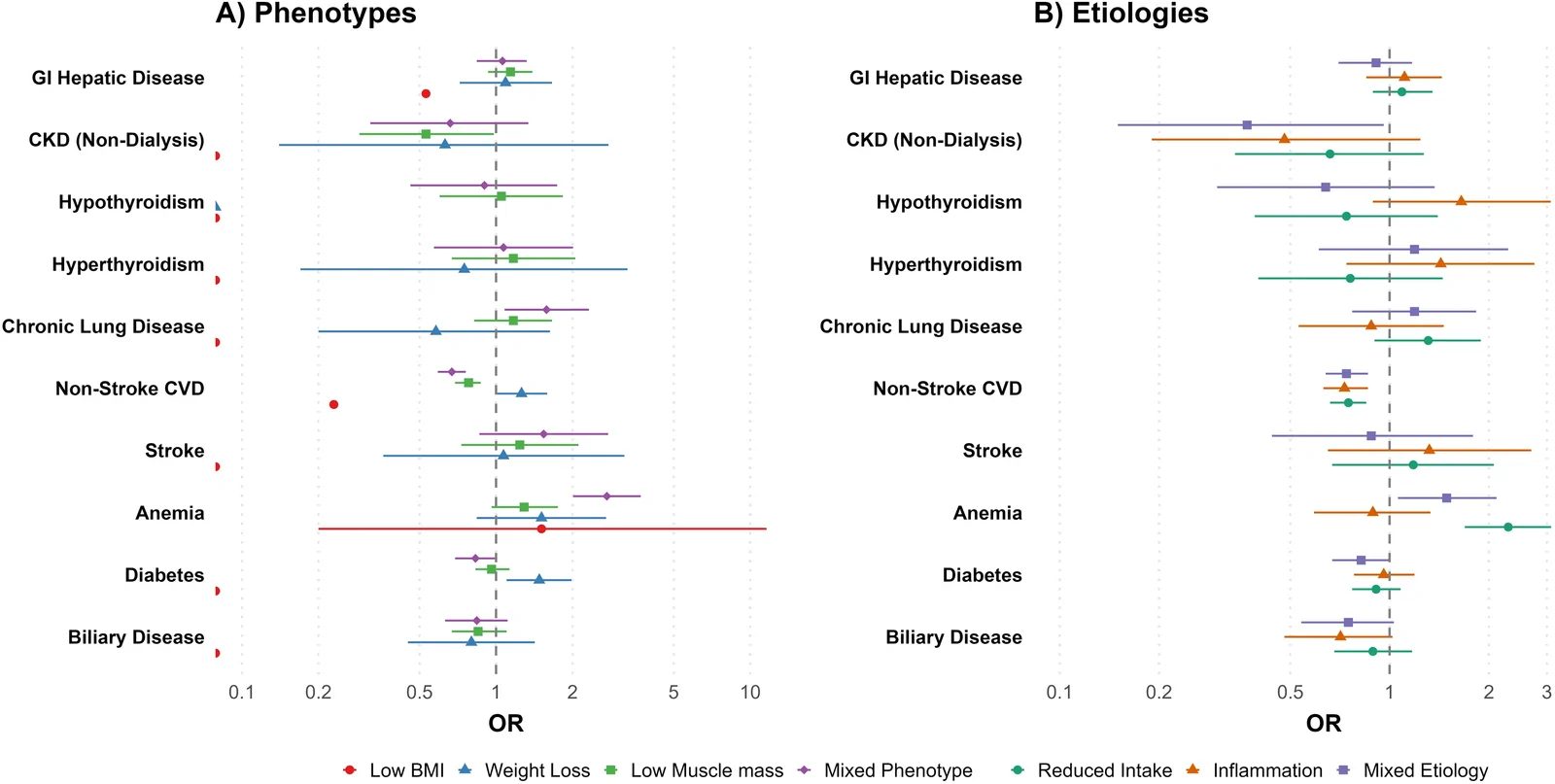
Adjusted association of comorbidities with the phenotypic and etiologic characteristics of GLIM-defined malnutrition. The forest plots display adjusted ORs and 95% CIs from multivariable multinomial logistic regression models. (A) The association between comorbidities and phenotypic characteristics. (B) The association between comorbidities and etiologic characteristics. All models were adjusted for age, sex, cancer site, cancer stage, and treatment. The well-nourished group served as the reference category for all analyses. Specific ORs and 95% CIs are in Table S4 and Table S5. Abbreviations: OR, Odds Ratio; BMI, Body Mass Index; CI, Confidence Interval; CKD, Chronic Kidney Disease; CVD, Cardiovascular Disease; GI, Gastrointestinal.

A heatmap was used to visually explore the association between specific comorbidities and characteristics of GLIM-defined malnutrition under univariate models (Figure S2). Subsequently, we used multivariable multinomial logistic regression models to further examine the above visual associations (Fig. 1). The results showed that, compared with the well-nourished group, anemia was significantly associated with an increased risk of reduced food intake/assimilation (OR = 2.29, 95% CI: 1.69–3.09), mixed etiology profile (OR = 1.49, 95% CI: 1.06–2.11), and mixed phenotype profile (OR = 2.73, 95% CI: 2.01–3.71). Chronic lung disease was associated with increased risk of mixed phenotype (OR = 1.58, 95% CI: 1.08–2.32). In contrast, non-stroke cardiovascular disease was associated with decreased risk of low BMI (OR = 0.23, 95% CI: 0.12−0.44), low muscle mass (OR = 0.78, 95% CI: 0.63−0.96), and mixed phenotype (OR = 0.34, 95% CI: 0.21−0.54). Non-dialysis chronic kidney disease was associated with decreased risk of low muscle mass (OR = 0.53, 95% CI: 0.29−0.98). Notably, while diabetes was associated with increased risk of weight loss (OR = 1.48, 95% CI: 1.10–1.98), it was also associated with decreased risk of mixed phenotype (OR = 0.83, 95% CI: 0.69−0.99). We did not observe independent associations of gastrointestinal/hepatic disease, thyroid disorders, or stroke and any phenotypic or etiologic characteristic.

### 3.4 Associations of comorbidity burden and malnutrition with clinical outcomes

#### 3.4.1 Independent prognostic value of comorbidity burden and malnutrition

**Fig. 2:**
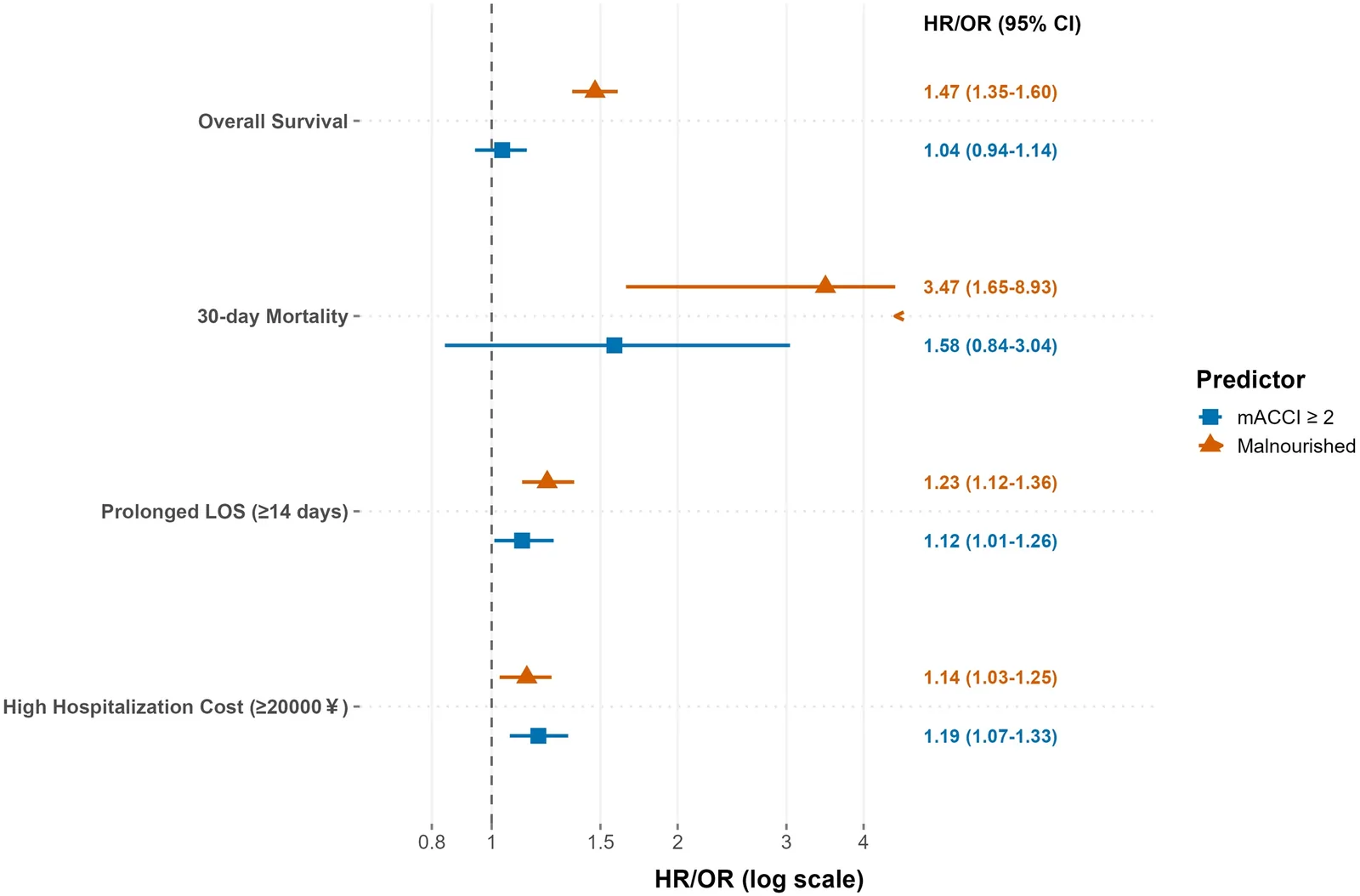
Independent associations of comorbidity burden and malnutrition with outcomes. Forest plot showing the adjusted HRs for Overall Survival and adjusted ORs for clinical outcomes. All models were adjusted for age, sex, cancer site, cancer stage, and treatment. Tests for interaction between comorbidity burden and malnutrition were performed for all outcomes; no statistically significant interaction was observed (all *p* > 0.05). Abbreviations: OR, Odds Ratio; HR, Hazard Ratio; CI, Confidence Interval; LOS, Length of Stay; mACCI, modified Age-adjusted Charlson Comorbidity Index.

In multivariate analyses, GLIM-defined malnutrition was an independent predictor of all four adverse clinical outcomes. Specifically, it was associated with poorer overall survival (HR = 1.52, 95% CI: 1.10–2.11), 30-day mortality (OR = 3.47, 95% CI: 1.65–8.93), prolonged LOS (OR = 1.23, 95% CI: 1.01–1.50), and increased hospitalization costs (OR = 1.17, 95% CI: 1.01–1.37) (Fig. 2). Similarly, a high comorbidity burden independently predicted prolonged LOS (OR = 1.12, 95% CI: 1.01–1.26) and increased hospitalization costs (OR = 1.19, 95% CI: 1.07–1.33). However, the independent associations of comorbidity burden with OS and 30-day mortality were not statistically significant. Furthermore, no statistically significant interactions between comorbidity burden and malnutrition were observed for any of the clinical outcomes (all interaction *p* >  0.05). The corresponding results remained similar in sensitivity analyses (Table S6).

#### 3.4.2 Additive prognostic value of comorbidity burden and malnutrition

**Fig. 3:**
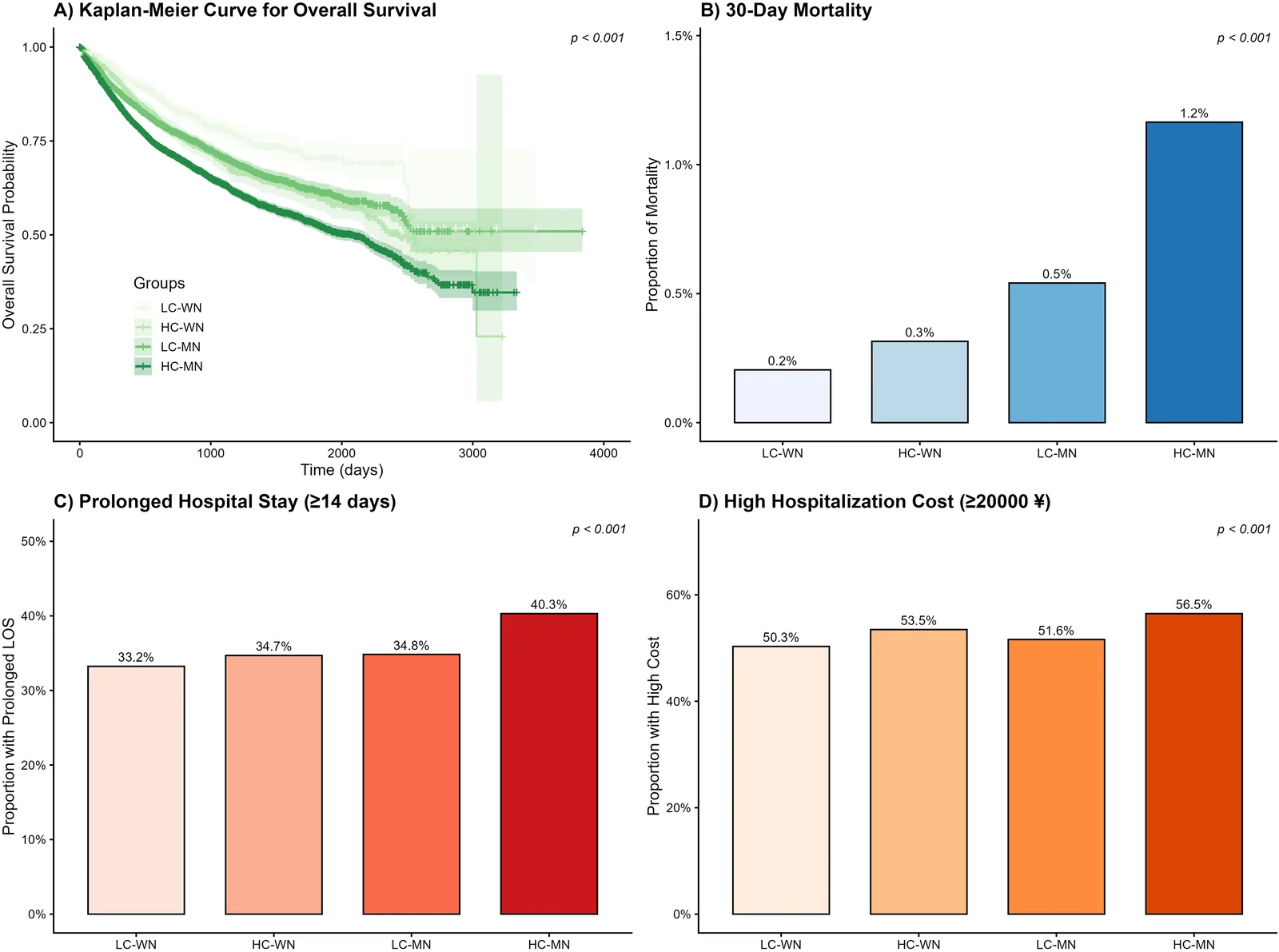
Combined associations of comorbidity burden and nutritional status with outcomes. (A) Overall survival probability observed across the four groups (log-rank test, *p* < 0.001). (B–D) Observed proportions of 30-day mortality, prolonged LOS (≥14 days), and increased hospitalization costs (≥20,000 ¥) across the four groups (chi-square test, *p* < 0.001). Abbreviations: LC-WN: low comorbidity burden and well-nourished (reference group); HC-WN: high comorbidity burden and well-nourished; LC-MN: low comorbidity burden and malnourished; HC-MN: high comorbidity burden and malnourished; LOS: Length of Stay.

Survival curve analysis (Fig. 3A) revealed significant differences in OS among the four groups (log-rank *p* < 0.001), with the high comorbidity burden and malnourished group (HC-MN) demonstrating the shortest OS. The incidence of adverse clinical events (Figs. 3B-D) increased progressively with the accumulation of comorbidity burden and malnutrition.

Multivariable analyses further confirmed this additive effect (Table 3). The high comorbidity burden and malnourished group (HC-MN) had the highest risk for prolonged LOS (OR = 1.33, 95% CI: 1.12–1.58), increased costs (OR = 1.35, 95% CI: 1.14–1.60), and a significantly elevated risk of 30-day mortality (OR = 4.58, 95% CI: 1.30–29.1).

**Table 3:** Multivariable analysis of the combined association of comorbidity and malnutrition with outcomes.

Outcome	Comorbidity burden and Nutrition Groups	HR/OR	95% CI	P-value
**Overall Survival (HR)**				
	LC-WN	Ref.	Ref.	Ref.
	HC-WN	1.13	0.94, 1.36	0.215
	LC-MN	1.46	1.25, 1.70	**<0.001**
	HC-MN	1.41	1.20, 1.67	**<0.001**
**30-Day Mortality (OR)**				
	LC-WN	Ref.	Ref.	Ref.
	HC-WN	1.25	0.23, 9.37	0.803
	LC-MN	2.97	0.86, 18.6	0.142
	HC-MN	4.58	1.30, 29.1	**0.044**
**Prolonged LOS (≥14d) (OR)**				
	LC-WN	Ref.	Ref.	Ref.
	HC-WN	1.05	0.86, 1.29	0.597
	LC-MN	1.15	0.99, 1.35	0.075
	HC-MN	1.33	1.12, 1.58	**0.001**
**Increased Cost (≥**20000**¥) (OR)**				
	LC-WN	Ref.	Ref.	Ref.
	HC-WN	1.17	0.96, 1.42	0.121
	LC-MN	1.17	1.01, 1.36	**0.039**
	HC-MN	1.35	1.14, 1.60	**<0.001**

All models were adjusted for age, sex, cancer site, cancer stage, and treatment.

Abbreviations: CI: Confidence Interval; HR: Hazard Ratio; OR: Odds Ratio; LC-WN: low comorbidity burden and well-nourished (reference group); HC-WN: high comorbidity burden and well-nourished; LC-MN: low comorbidity burden and malnourished; HC-MN: high comorbidity burden and malnourished.

## 4 Discussion

To our knowledge, this multicenter prospective cohort study is the first to systematically evaluate the relationship between comorbidity and malnutrition in cancer patients. We found that a high comorbidity burden was present in 62.0% of cancer patients. The association between comorbidity burden and malnutrition was stage-dependent and only showed a statistical significance among patients in stage I. Some comorbidities exhibited distinct associations with specific phenotypic and etiologic characteristics of malnutrition. In addition, the coexistence of the two factors had an additive effect on clinical outcomes. These findings not only reveal the complex relationship between comorbidity and malnutrition, but also provide important epidemiological evidence for integrating comorbidity assessment into nutrition management to achieve more precise prognostic risk stratification.

In this cohort, the prevalence of malnutrition was 82.4%, which was at the upper end of previously reported ranges [2]. This might be due to the fact that this study included a high proportion of patients with gastrointestinal cancer and advanced cancer, as well as the direct application of the GLIM criteria for diagnosing malnutrition, which avoided potential underdiagnosis caused by screening. In this context, our study yielded three key findings.

One of the key findings is that the relationship between comorbidity burden and malnutrition is stage-dependent. After adjusting the key covariates, the significant correlation only occurred in stage I patients. This is consistent with previous studies where the risk factors that play a major role in nutritional status change with the cancer progression [30]. For patients with early-stage cancer, lower tumor burden has little impact on metabolism [31]. On the one hand, inflammation, metabolic disorders, and organ dysfunction accompanying comorbidities may become the main factors affecting nutrition [10,11]. As cancer progresses, severe metabolic disorders and cachexia driven by malignancy may overshadow the impact of comorbidity [32,33]. On the other hand, the correlation between the two disappeared statistically in patients with advanced stage, which may be caused by residual confounding and insufficient statistical power. Our findings suggest that, for patients with early cancer, nutritional assessment should not only be limited to the tumor itself, but also take the comorbidity burden into account.

Secondly, this study revealed association patterns between different comorbidities and phenotypic/etiologic characteristics of malnutrition. Anemia was significantly positively correlated with multiple phenotypic characteristics of malnutrition (such as weight loss and low muscle mass). Malnutrition can directly cause nutritional anemia [34], while anemia-related fatigue and appetite loss may worsen the nutritional status [35]. In addition, this association may stem from chronic inflammation as a common upstream driving factor. Systemic inflammation can not only drive cancer cachexia but also induce chronic anemia through the mechanism mediated by hepcidin [36]. In contrast, non-stroke cardiovascular disease and non-dialysis chronic kidney disease were negatively correlated with phenotypic characteristics of malnutrition, such as low BMI and low muscle mass. This seemingly paradoxical finding highlights the complexity of clinical assessment. First, cardiovascular and renal diseases are usually accompanied by fluid retention [37,38]. This may maintain or increase body weight and limb circumference, thereby masking true tissue loss. Second, there may be reverse causality or survivor bias. This means that patients with severe cardiovascular or renal disease who survived to be enrolled in the cohort may have protective characteristics not measured in this study, such as a higher baseline BMI [39]. These findings suggest that, for cancer patients with these comorbidities, relying only on BMI or muscle mass index may underestimate the risk of malnutrition. This highlights the need for tools that can distinguish between fluid and tissue, such as bioelectrical impedance analysis (BIA). At the same time, we should also pay attention to the evaluation of weight history and dietary intake.

In addition, chronic lung disease was associated with an increased risk of mixed phenotypes of malnutrition. This is likely related to increased energy consumption from elevated work of breathing, systemic inflammation, and the use of glucocorticoids [40]. Diabetes and malnutrition showed a double correlation. On the one hand, it is associated with an increased risk of weight loss, which may be caused by pathological processes such as glucose metabolism disorders, catabolism, and osmotic diuresis in diabetic patients [41]. On the other hand, diabetes is associated with a reduced risk of mixed phenotypes. It may be due to the spurious association caused by the generally high baseline BMI of patients with type 2 diabetes [42]. In our study, patients with diabetes also had a significantly higher BMI than those without diabetes (23.4 vs. 22.4, *p* < 0.01) in our study. This finding suggests that relying solely on BMI for nutritional assessment in patients with cancer and concomitant diabetes may lead to a missed diagnosis of malnutrition. Therefore, non-BMI indices should be employed. Finally, some chronic diseases, such as gastrointestinal and liver diseases, were not associated with malnutrition. This does not negate their importance but may suggest that the anorexia and inflammatory effects caused by cancer and its treatment may mask the influence of these comorbidities. Overall, these findings indicate the need to shift from the macroscopic concept of comorbidity burden to a microscopic analysis of specific comorbidities for personalized nutritional assessment.

Consistent with previous research [4,5], this study confirmed that malnutrition is an independent prognostic factor for all four adverse outcomes. High comorbidity burden is primarily associated with prolonged LOS and increased hospitalization costs, reflecting greater healthcare resource consumption [43]. We also found that combining comorbidity burden and malnutrition improved prognostic stratification. Specifically, a clear accumulation of risk was observed for 30-day mortality. Although the limited number of 30-day deaths reduces the precision of the results, the trend of increasing relative risk is observable and aligned with the results of Shao et al., who similarly found that the combination of comorbidity burden and sarcopenia/body composition can improve the ability to discriminate the prognosis in post-operative gastric cancer patients [13,44]. This further indicates that cancer patients with both high comorbidity burden and malnutrition may constitute a high-risk subgroup with poor prognosis, which requires urgent attention. For these patients, a higher-priority, multidisciplinary, collaborative approach integrating oncology, nutrition, and comorbidity management may be required to improve prognosis.

This study has several limitations. First, as detailed in methods, for the purpose of improving the accuracy of malnutrition diagnosis and enhancing the internal validity of this study, we chose to directly apply the GLIM criteria. However, this design might result in the estimated prevalence of malnutrition being higher than that of studies that followed the two-step screening method, thus limiting the generalizability of prevalence estimates. The trade-off between maximizing internal validity for research and ensuring external validity for prevalence estimation highlights a critical consideration for future studies in this field. Second, low muscle mass was assessed using anthropometric measures, including CC and MAMC with fixed sex-specific cut-offs rather than BMI-adjusted thresholds. The accuracy of these measures might be affected by subcutaneous fat in patients with high BMI or fluid retention in edematous patients [37,38], leading to potential overestimation of muscle mass and underestimation of malnutrition in some patients. However, using validated anthropometric indices is a widely accepted alternative approach in large-scale epidemiological surveys where more precise measurement tools are unavailable [45]. More precise techniques like BIA are needed to validate these findings. Third, although the mACCI is a widely applied standardized tool, it cannot fully capture the severity and complexity of comorbidities. The range of comorbidities investigated in this study was relatively narrow. These may underestimate the actual comorbidity burden. Future research could assess comorbidity more accurately by integrating more objective indicators (such as laboratory data and disease classification) from electronic medical records. Finally, this was an observational study. Apart from the survival-related prognostic analyses, the other findings reflect correlation rather than causality. They provide crucial epidemiological evidence for future intervention studies.

## 5 Conclusion

In summary, comorbidity burden is a stage-dependent risk factor for malnutrition in cancer patients, and different comorbidities exhibit unique patterns of association with phenotypic/etiologic characteristics of malnutrition. In addition, the combination of comorbidity burden and malnutrition may improve prognostic stratification in cancer patients. These findings suggest that information related to comorbidities should be taken into account in the nutritional management of cancer patients.

## Authorship

**Kaijia Zhao**: Conceptualization, Methodology, Data curation, Formal analysis, Writing - original draft. **Huanyu Hu and Hongzhen Du**: Validation, Investigation, Writing - original draft. **Caiyan Zhang, Yeze Jia, Xiaoya Wang, Xuanhui Wu, Yan Guo, Yijing Zhai, Mingyang Fu, and Anqi Wang**: Data curation, Investigation. **Zengning Li**: Conceptualization, Funding acquisition, Project administration, Writing - review & editing, Supervision. **Hanping Shi, and Chunhua Song**: Conceptualization, Project administration, Writing - review & editing, Supervision. All authors revising it and approved the final manuscript.

## Declaration of Generative AI and AI-assisted technologies in the writing process

During the preparation of this manuscript, the authors used Gemini to improve language and readability. After using this tool, the authors reviewed and edited the content as needed and take full responsibility for the content of the publication.

## Funding

This research was supported by National Natural Science Foundation of China (Grant No. 82373550)

## Data availability

The datasets analyzed during this study are available from the corresponding author on reasonable request.

## Declaration of competing interest

The authors declare no conflicts of interest.
