# Supplementary material for: Comorbidity, malnutrition, and their additive effect on prognosis in cancer patients: a multicenter cohort study
Source: J Nutr Health Aging. 2026 Aug 25;30(10):100960. doi: 10.1016/j.jnha.2026.100960 (PMC13543010; doi:10.1016/j.jnha.2026.100960)
Supplement: Supplementary file 1 [file mmc1.docx]

**Figure S1. Flow chart of participants**

**Table S1. Weighted index of comorbidities and age**

| **Condition** | **Weight** |
| --- | --- |
| Diabetes | 1 |
| Hypertension | 1 |
| Heart disease | 1 |
| Gastrointestinal or digestive system disease | 1 |
| Chronic lung disease | 1 |
| Kidney disease | 2 |
| Stroke | 1 |
| Liver disease |  |
| chronic hepatitis | 1 |
| cirrhosis | 3 |
| Connective tissue disease | 1 |
| Age (years old) |  |
| 50~59 | 1 |
| 60~69 | 2 |
| 70~79  80~ | 3  4 |

**Table S2. The GLIM criteria defined in this study**

| **Phenotypical criteria** | | |  | **Etiological criteria** | |
| --- | --- | --- | --- | --- | --- |
| **Non-volitional weight loss** | **Low BMI (kg/m^2^)** | **Low muscle mass** |  | **Reduced food intake or assimilation** | **Disease burden/inflammation** |
| >5% within the past 6 months  **OR**  >10% beyond 6 months | < 70 years:  BMI<18.5,  ≥ 70 years:  BMI<20 | **CC** [1]:  Men: <34cm,  women: < 33 cm  **OR**  **MAMC** [2]:  Men: <23cm,  women: < 22 cm |  | 50% of energy requirements for >1 week, any reduction for >2 weeks  **OR**  any chronic gastrointestinal condition that adversely affects food assimilation or absorption | Acute disease/injury with acute inflammation of a severe degree (e.g., major infections, burns, trauma, and closed head injury)  **OR**  Chronic disease with chronic or recurrent inflammation of a mild to moderate degree (e.g., congestive heart failure, chronic obstructive pulmonary disease, rheumatoid arthritis, chronic kidney or liver disease, and cancer)  **OR**  Indicators such as CRP (> 3 mg/L), NLR (> 3), and body temperature (> 38 ℃) were used as supportive proxy measures of inflammation. |

Abbreviations: GLIM: The Global Leadership Initiative on Malnutrition; BMI: body mass index; CC: calf circumference; MAMC: Mid-Arm Muscle Circumference; CRP: C-Reactive Protein; NLR: Neutrophil-to-Lymphocyte Ratio.

**Table S3. Multivariable and subgroup analyses of the association between comorbidity burden and malnutrition**

| Characteristic | Overall population  (*N*=12947) | | |  | Subgroup analysis by cancer stage  (using Model 4) | | | |
| --- | --- | --- | --- | --- | --- | --- | --- | --- |
|  | Model 1 | Model 2 | Model 3 |  | Stage I  (*n*=1891) | Stage II  (*n*=1537) | Stage III  (*n*=3641) | Stage IV  (*n*=5878) |
| Comorbidity Burden,  OR (95% CI) |  |  |  |  |  |  |  |  |
| mACCI＜2 | Ref. | Ref. | Ref. |  | Ref. | Ref. | Ref. | Ref. |
| mACCI≥2 | 1.30  (1.19, 1.43) | 1.39  (1.27, 1.53) | 1.42  (1.29, 1.56) |  | 2.11  (1.65, 2.69) | 1.79  (1.34, 2.39) | 1.32  (1.11, 1.57) | 1.24  (1.07, 1.43) |
| P-value | **<0.001** | **<0.001** | **<0.001** |  | **<0.001** | **<0.001** | **0.002** | **0.003** |

Model 1: Crude model.

Model 2: Adjusted for sex.

Model 3: Adjusted for sex, cancer site, cancer stage, and cancer-related treatment.

Model 4: Adjusted for sex, cancer site, and cancer-related treatment.

*p* for interaction between Comorbidity Burden (mACCI ≥2) and Cancer Stage = 0.005

Table S4. Association of comorbidities with the phenotypic characteristics of GLIM-defined malnutrition

| **Comorbidity** | Low BMI  OR (95% CI) | Mixed Phenotype  OR (95% CI) | Muscle Loss  OR (95% CI) | Weight Loss  OR (95% CI) | **p-value** |
| --- | --- | --- | --- | --- | --- |
| GI_Hepatic_Disease | 0.53(0.07, 3.99) | 1.06(0.84, 1.32) | 1.14(0.93, 1.39) | 1.09(0.72, 1.66) | 0.6 |
| Kidney_Disease_Stage |  |  |  |  | 0.2 |
| CKD (Non-Dialysis) | 0.00(0.00, 0.00) | 0.66(0.32, 1.34) | **0.53(0.29, 0.98)** | 0.63(0.14, 2.77) |  |
| Dialysis | 8.54(8.54, 8.54) | 46,002(23,398, 90,442) | 71,794(36,517, 141,153) | 0.10(0.10, 0.10) |  |
| Thyroid_Status |  |  |  |  | 0.5 |
| Hypothyroidism | 0.00(0.00, 0.00) | 0.90(0.46, 1.74) | 1.05(0.60, 1.83) | 0.00(0.00, 0.00) |  |
| Hyperthyroidism | 0.00(0.00, 0.00) | 1.07(0.57, 2.01) | 1.17(0.67, 2.05) | 0.75(0.17, 3.29) |  |
| Chronic_Lung_Disease | 0.00(0.00, 0.00) | **1.58(1.08, 2.32)** | 1.17(0.82, 1.66) | 0.58(0.20, 1.63) | **0.030** |
| Non_Stroke_CVD | **0.23(0.07, 0.79)** | **0.67(0.59, 0.76)** | **0.78(0.69, 0.87)** | 1.26(0.99, 1.59) | **<0.001** |
| Stroke | 0.00(0.00, 0.00) | 1.54(0.86, 2.76) | 1.24(0.73, 2.11) | 1.07(0.36, 3.20) | 0.6 |
| Anemia | 1.51(0.20, 11.6) | **2.73(2.01, 3.71)** | 1.29(0.96, 1.75) | 1.51(0.84, 2.71) | **<0.001** |
| Diabetes | 0.00(0.00, 0.00) | **0.83(0.69, 0.99)** | 0.96(0.83, 1.13) | **1.48(1.10, 1.98)** | **<0.001** |
| Biliary_Disease | 0.00(0.00, 0.00) | 0.84(0.63, 1.11) | 0.85(0.67, 1.10) | 0.80(0.45, 1.42) | 0.4 |

Abbreviations: CI = Confidence Interval, OR = Odds Ratio. CKD, Chronic Kidney Disease; CVD, Cardiovascular Disease; GI, Gastrointestinal.

Extremely large ORs (e.g., OR > 40,000 for Dialysis) indicate quasi-complete separation in the model and should be interpreted as strong, but imprecise associations.

Table S5. Association of comorbidities with the etiologic characteristics of GLIM-defined malnutrition

| **Comorbidity** | Inflammation-Driven  OR (95% CI) | Intake-Driven  OR (95% CI) | Mixed-Driven  OR (95% CI) | **p-value** |
| --- | --- | --- | --- | --- |
| GI_Hepatic_Disease | 1.11(0.85, 1.44) | 1.09(0.89, 1.35) | 0.91(0.70, 1.17) | 0.3 |
| Kidney_Disease_Stage |  |  |  | 0.11 |
| CKD (Non-Dialysis) | 0.48(0.19, 1.24) | 0.66(0.34, 1.27) | **0.37(0.15, 0.96)** |  |
| Dialysis | 30,461(10,263, 90,411) | 25,219(10,381, 61,262) | 23,062(7,851, 67,745) |  |
| Thyroid_Status |  |  |  | **0.020** |
| Hypothyroidism | 1.65(0.89, 3.08) | 0.74(0.39, 1.40) | 0.64(0.30, 1.37) |  |
| Hyperthyroidism | 1.43(0.74, 2.75) | 0.76(0.40, 1.45) | 1.19(0.61, 2.29) |  |
| Chronic_Lung_Disease | 0.88(0.53, 1.46) | 1.31(0.90, 1.89) | 1.19(0.77, 1.83) | 0.3 |
| Non_Stroke_CVD | **0.73(0.63, 0.86)** | **0.75(0.66, 0.85)** | **0.74(0.64, 0.86)** | **<0.001** |
| Stroke | 1.32(0.65, 2.69) | 1.18(0.67, 2.07) | 0.88(0.44, 1.79) | 0.7 |
| Anemia | 0.89(0.59, 1.33) | **2.29(1.69, 3.09)** | **1.49(1.06, 2.11)** | **<0.001** |
| Diabetes | 0.96(0.78, 1.19) | 0.91(0.77, 1.08) | 0.82(0.67, 1.00) | 0.2 |
| Biliary_Disease | 0.71(0.48, 1.02) | 0.89(0.68, 1.17) | 0.75(0.54, 1.03) | 0.11 |

Abbreviations: CI = Confidence Interval, OR = Odds Ratio. CKD, Chronic Kidney Disease; CVD, Cardiovascular Disease; GI, Gastrointestinal.

Extremely large ORs (e.g., OR > 40,000 for Dialysis) indicate quasi-complete separation in the model and should be interpreted as strong, but imprecise associations.

**Figure S2. Exploratory associations of comorbidities with the phenotype and etiology profiles of GLIM-defined malnutrition**

The heatmaps illustrate the results of univariate multinomial logistic regression analyses. A) The association between comorbidities (rows) and the four phenotype profiles (columns). B) The association between comorbidities (rows) and the three etiology profiles (columns).

Cell values represent Odds Ratios (ORs) from the corresponding univariate models. Red indicates a positive association (OR > 1) and blue indicates a negative association (OR < 1), with the Well-nourished patients serving as the reference group.

Abbreviations: GLIM, Global Leadership Initiative on Malnutrition; CKD, Chronic Kidney Disease; CVD, Cardiovascular Disease; GI, Gastrointestinal.

* *p* < 0.05, ** *p* < 0.01, *** *p* < 0.001

**Table S6. Effects of comorbidity burden, nutritional status, and their interaction on outcomes: multi-model regression analyses**

|  | Model 1: Crude Model  HR/OR (95% CI) | Model 2: Adjusted Model  HR/OR (95% CI) | Model 3: Interaction Model  HR/OR (95% CI) |
| --- | --- | --- | --- |
| Overall Survival (HR) |  |  |  |
| mACCI | 1.12(1.10, 1.15)** | 1.01(0.97, 1.04) | 1.01(0.96, 1.07) |
| Malnutrition | 1.47(1.34, 1.60)** | 1.47(1.35, 1.61)** | 1.50(1.29, 1.75)** |
| mACCI × Malnutrition |  |  | 0.99(0.94, 1.05) |
| 30-day Mortality (OR) |  |  |  |
| mACCI | 1.33(1.19, 1.50)** | 1.34(1.12, 1.58)** | 1.42(0.83, 2.28) |
| Malnutrition | 3.52(1.68, 9.02)** | 3.52(1.67, 9.05)** | 4.16(1.03, 24.4) |
| mACCI × Malnutrition |  |  | 0.94(0.58, 1.59) |
| Prolonged LOS (OR) |  |  |  |
| mACCI | 1.08(1.05, 1.11)** | 1.05(1.01, 1.10)* | 1.00(0.94, 1.08) |
| Malnutrition | 1.20(1.09, 1.32)** | 1.23(1.12, 1.36)** | 1.10(0.93, 1.30) |
| mACCI × Malnutrition |  |  | 1.06(0.99, 1.14) |
| increased Hospitalization Cost (OR) |  |  |  |
| mACCI | 1.08(1.05, 1.10)** | 1.09(1.05, 1.14)** | 1.09(1.02, 1.17)* |
| Malnutrition | 1.11(1.01, 1.21)* | 1.14(1.04, 1.26)** | 1.14(0.97, 1.33) |
| mACCI × Malnutrition |  |  | 1.00(0.94, 1.07) |

Abbreviations: OR = Odds Ratio; HR = Hazard Ratio; CI = Confidence Interval; LOS = Length of Stay; mACCI = modified Age-adjusted Charlson Comorbidity Index.

Model 1: Crude model.

Model 2 and Model 3 were adjusted for age, sex, cancer site, cancer stage, and cancer-related treatment.

Model 2 includes main effects of mACCI and malnutrition. Model 3 includes main effects of mACCI and malnutrition, and their interaction term.

**p* < 0.05, ***p* < 0.01

## References

[1] Chen LK, Woo J, Assantachai P, Auyeung TW, Chou MY, Iijima K, et al. Asian Working Group for Sarcopenia: 2019 Consensus Update on Sarcopenia Diagnosis and Treatment. J AM MED DIR ASSOC. 2020, 21(3): 300-7.

[2] Hanping Shi, Wenhua Ling, Zengning Li. Clinical Nutrition (in Chinese). Beijing, China: People's Medical Publishing House. 2022: 135.
